# Promoting inclusion in clinical trials—a rapid review of the literature and recommendations for action

**DOI:** 10.1186/s13063-021-05849-7

**Published:** 2021-12-04

**Authors:** Danielle H. Bodicoat, Ash C. Routen, Andrew Willis, Winifred Ekezie, Clare Gillies, Claire Lawson, Thomas Yates, Francesco Zaccardi, Melanie J. Davies, Kamlesh Khunti

**Affiliations:** 1 Independent Researcher, Leicester, UK; 2 Centre for Ethnic Health Research, University of Leicester, Leicester General Hospital, Leicester, UK; 3 Diabetes Research Centre, University of Leicester, Leicester General Hospital, Leicester, UK; 4 NIHR Leicester Biomedical Research Centre, Leicester General Hospital, Leicester, UK; 5 Leicester Real World Evidence Unit, Diabetes Research Centre, University of Leicester, Leicester General Hospital, Leicester, UK; 6 Leicester Diabetes Centre, University Hospitals of Leicester NHS Trust, Leicester, UK

**Keywords:** Equality, Diversity, Inclusion, Ethnicity, Clinical research, Clinical trial, Review

## Abstract

**Background:**

Without inclusion of diverse research participants, it is challenging to understand how study findings will translate into the real world. Despite this, a lack of inclusion of those from under-served groups in research is a prevailing problem due to multi-faceted barriers acting at multiple levels. Therefore, we rapidly reviewed international published literature, in relation to clinical trials, on barriers relating to inclusion, and evidence of approaches that are effective in overcoming these.

**Methods:**

A rapid literature review was conducted searching PubMed for peer-reviewed articles that discussed barriers to inclusion or strategies to improve inclusion in clinical trial research published between 2010 and 2021. Grey literature articles were excluded.

**Results:**

Seventy-two eligible articles were included. The main barriers identified were language and communication, lack of trust, access to trials, eligibility criteria, attitudes and beliefs, lack of knowledge around clinical trials, and logistical and practical issues. In relation to evidence-based strategies and enablers, two key themes arose: [1] a multi-faceted approach is essential [2]; no single strategy was universally effective either within or between trials. The key evidence-based strategies identified were cultural competency training, community partnerships, personalised approach, multilingual materials and staff, communication-specific strategies, increasing understanding and trust, and tackling logistical barriers.

**Conclusions:**

Many of the barriers relating to inclusion are the same as those that impact trial design and healthcare delivery generally. However, the presentation of these barriers among different under-served groups may be unique to each population’s particular circumstances, background, and needs. Based on the literature, we make 15 recommendations that, if implemented, may help improve inclusion within clinical trials and clinical research more generally. The three main recommendations include improving cultural competency and sensitivity of all clinical trial staff through training and ongoing personal development, the need to establish a diverse community advisory panel for ongoing input into the research process, and increasing recruitment of staff from under-served groups. Implementation of these recommendations may help improve representation of under-served groups in clinical trials which would improve the external validity of associated findings.

**Supplementary Information:**

The online version contains supplementary material available at 10.1186/s13063-021-05849-7.

## Background

The importance of inclusion in health and social care research has gained increasing recognition, as further highlighted in the COVID-19 pandemic [1]. Without participants from a broad range of backgrounds (age, gender, ethnicity, comorbidities), it is not possible to understand how study findings will translate into real-world application, and this is particularly true for clinical trials.

The term under-served group refers to segments of the population who are represented in health research at lower levels than would be expected from population estimates [2]. Groups considered under-served [2] in clinical research are heterogenous and are often crudely considered in terms of basic characteristics, such as ethnicity, disability, or age. But what constitutes under-served is complex and context-specific—and may be disease or study-specific [2]. General examples of under-served groups are often defined by demographic, social, or economic factors; health factors; and/or disease-specific characteristics [2]. Working-age populations, for example, are often under-served in research, despite not typically being considered as under-served in other settings. Despite the ethical and scientific implications of a lack of inclusion in research, it remains a widespread issue [3–6], as demonstrated in recent COVID-19-specific trials [7].

For example, ethnic minority involvement in health and social care research mostly occurs during the research design phase and least in data analysis and interpretation [8]. The majority of evidence on ethnic minority inclusion in clinical research is from the USA, with relatively little other research globally [9]. Furthermore, defining the true scale of the issue is made difficult by a lack of reporting relating to protected characteristics. For example, a systematic review found that of 1518 COVID-19-related studies registered on ClinicalTrials.gov, only six reported collecting data on ethnicity [10]. There are numerous inter-related reasons for the lack of diversity among clinical research participants, not least, that recognition and acceptance of the issue is relatively new compared with long-standing practices of research. Barriers to successful inclusion (i.e. increased representation of under-served groups in clinical trial populations) can broadly be considered to coalesce into issues relating to communication between researchers and participants, how trials are designed and delivered, differing agendas of research teams and participant groups, and a lack of trust in the research process [11, 12].

Given the COVID-19 pandemic has demonstrated the necessity of improving inclusion in clinical research and the UK government’s call for increased diversity in clinical research, there is a pressing need for understanding and education around barriers to inclusion in clinical research and improvement strategies. In particular, the current and ongoing COVID-19 trials require up-to-date and actionable information on how to improve inclusion in their cohorts. Therefore, there is a distinct necessity for a rapid review of the literature on barriers and enablers of inclusion in clinical research.

Consequently, the primary purpose of this paper was to review international published literature and produce a high-level summary of existing evidence and studies that consider the specific barriers in relation to inclusion in clinical trials and evidence of approaches that are effective in overcoming these. A second aim was to make recommendations for how clinical researchers can support trials to be diverse and inclusive.

## Methods

### Design

A rapid review is a method of knowledge synthesis that accelerates the process of conducting a systematic review, by simplifying or omitting various stages of the process (e.g. search terms and inclusion criteria, data extraction, and bias assessment). This streamlining of the traditional systematic review methodology permits the production of evidence synthesis in a timely/resource-efficient manner for use by stakeholders. A rapid review was conducted over a 3-week period, from 13/03/2021 to 30/03/2021.

### Search strategy

A search of published literature was conducted in PubMed using the search terms detailed in Additional File 1. The search was limited to papers published in English language from 1 January 2010 onwards to ensure that the findings represented the contemporary research landscape. Reference lists of included articles were handsearched for additional relevant literature.

### Eligibility criteria, study selection, and quality assessment

The inclusion criteria included peer-reviewed articles that discussed barriers to inclusion or strategies to improve inclusion in clinical trial research published between January 2010 and March 2021. Grey literature articles were excluded.

Title and abstract screening of articles identified from electronic searches against the inclusion criteria was performed by a single reviewer (DHB). Full-text articles were also screened for inclusion by a single reviewer (DHB). Quality assessment of included articles was not undertaken to facilitate the rapid review of evidence.

### Data synthesis

The following data were extracted: first author, year, country, study type/design, number of trials, and number of participants (some studies reported both because they reported on their approaches over multiple trials), under-served groups considered, conditions considered (e.g. sleep, asthma, cancer, breastfeeding), and findings/information relating to enabling strategies or barriers. Findings from included articles were synthesised using tables and a narrative summary by a single reviewer (DHB), following a narrative, descriptive synthesis approach. Findings were summarised into two key themes relating to barriers to inclusion, and strategies used to overcome these, with a number of sub-themes within each overarching theme.

## Results

### Screening results

**Fig. 1:**
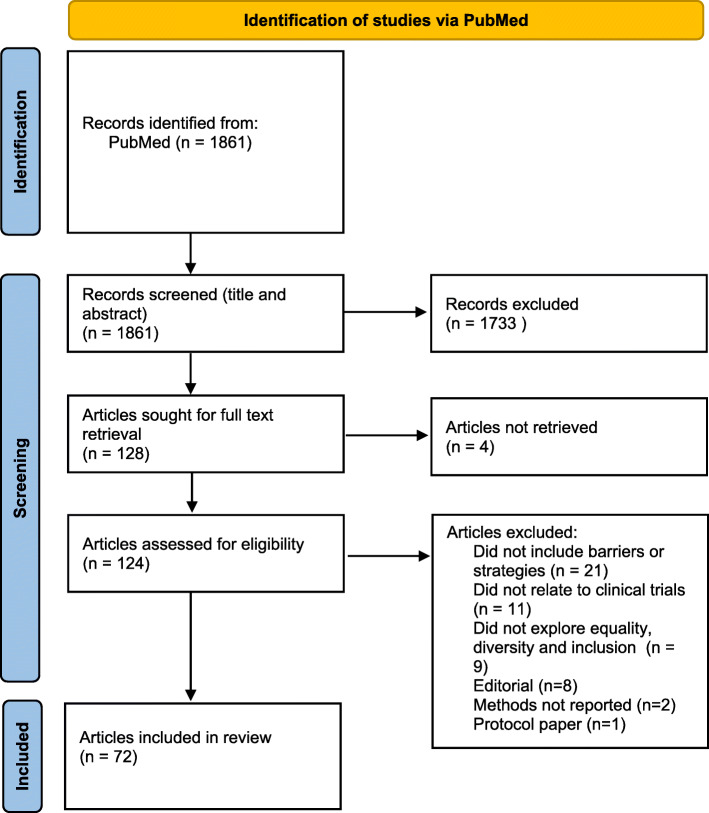
Screening and inclusion of identified articles

A total of 1861 results were returned from the search and screened for inclusion. Overall, 72 articles were included within this review (see Fig. 1 for screening data). The majority of the included articles (74%) were from the USA and a third focused on cancer trials (33%). In relation to the type of under-served groups included, half of all articles (50%) focused on inclusion issues relating to ethnic minority populations. The remainder focused on general under-served groups, children and young people, adolescents, women, and others (e.g. deaf, transgender). Sixty-one (85%—see Additional File 1 for more details) of the included articles reported on strategies to improve inclusion (of which *n* = 13 also included information on barriers). Eleven articles reported on barriers to inclusion alone. The 72 papers are included within the “Results” section, Additional File 1, or a combination of these.

### Barriers to inclusion

A consistent theme across the literature is that many of the barriers relating to inclusion are the same as those that impact trial design and delivery [13]. The nuance is around how these barriers present among different, under-served groups, which is often unique to each population’s particular circumstances, background, beliefs, and needs. Barriers are summarised below.

#### Language and communication

This well-known barrier to inclusion, particularly among recent migrant ethnic groups, remains an ongoing issue. In trials conducted in the USA and Europe, being unable to speak and/or read English is a common barrier faced by individuals [14]. Closely related to this is the ability to speak and/or read English to a certain level, without fully comprehending the meaning of everything that is said or written [15]. There is also the further issue of how this decision is made, i.e. who decides whether someone speaks good enough English to take part and what criteria are used. Importantly, language barriers can also be viewed as a failure to show respect to potential participants if the information has not been made available in a culturally relevant and accessible language format [16].

Finally, language/literacy barriers do not only apply to migrant ethnic groups, as other under-served populations also experience language/literacy barriers due to a range of issues such as disability or the impact of lack of education/access to education on literacy. For example, deaf individuals may need support with sign language [17, 18].

Poor communication was another common barrier that was predominantly identified in studies with groups [19, 20]. This may relate to literacy generally and also to health literacy more specifically [15]. Similarly, studies in children need age-appropriate communication [21].

#### Lack of trust

For potential participants, having a lack of trust in research, doctors, investigators, drugs, and the medical industry was a recurring theme across the literature [14–16, 19, 20, 22–24]. This may arise from previous bad experiences and previous severe adverse events in reported studies [25] and was often compounded by related beliefs or fears [23]. For example, older adults may fear that experimentation could damage their health [26] or that participation would not benefit younger generations [27].

#### Access to trials

A lack of access to relevant clinical trials manifested in a number of different ways. First, a lack of information about trials for which potential participants are eligible and available is a barrier [22, 23, 28]. This may particularly be the case for people without a usual place for care, who are also often not eligible for relevant trials [29]. Similarly, the inability to access the healthcare or research centre was a barrier [20, 28, 30]. Not being invited to eligible trials was another access-related barrier [31]. Finally, other practical factors preventing access to trials more generally remain important in under-served groups, such as recruitment competition for other studies and lack of recruitment staff [32].

#### Eligibility criteria

Some studies highlighted that inclusion/exclusion criteria often disproportionately exclude people in under-served groups, including older adults, pregnant women, obese individuals, people with existing/multiple chronic conditions (multi-morbidity), and people with severe mental illness [27, 28, 32]. This exclusion may be explicit; for example, lack of capacity to consent is a common exclusion criterion that means individuals without this capacity are denied the opportunity to participate in research [33]. It also means there is consequently a limited evidence base regarding health interventions in this population [33]. On the other hand, eligibility criteria may indirectly exclude some populations to a greater extent than others; for example, exclusion criterion may be more prevalent in some ethnic minority groups than in White Europeans, such as chronic diseases [34].

#### Attitudes and beliefs

This barrier can present in many different ways and is often context and populationspecific. Examples that arise in the literature include no personal or family history of the condition under study [29]; stigma surrounding the condition under study [13, 16, 24]; beliefs among older adults that they were too old to participate in trials [26]; concerns around immigration status for some ethnic minority populations [23]; concerns about side effects or taking an experimental medicine [27, 35]; stress, fatalism, and a conservative attitude to risk-taking among Asian women [25]; religious beliefs [30]; “Guinea pig” perceptions [36]; not feeling comfortable, welcome, or respected [36]; privacy concerns [23]; and negative attitudes to clinical trials [30]. In addition, not only the individual’s own beliefs and attitudes, but also those of their friends and families can prevent under-served groups from taking part in trials, as lack of social approval was found to be an important barrier [27, 28, 36, 37].

#### Lack of knowledge around clinical trials

There were a large number of studies that identified a lack of understanding about clinical trials as a barrier in a range of populations, particularly among ethnic minorities [14, 19, 22, 30]. Examples of where this lack of knowledge/information was apparent included the trial process such as during recruitment or the collection of data [22, 23].

#### Logistical and practical issues

Logistical and practical barriers were particularly prevalent among under-served groups studied [15], including lack of transport [16, 20, 28, 30, 32], time [16, 22, 24, 28], additional visits/tests [25], indirect costs associated with participating [23], childcare [20, 30], work responsibilities [20], and issues related to the condition under study, e.g. pregnancy or drug abstinence [24, 25, 28].

#### Other barriers

Aside from these key barriers that were commonly referred to across the literature, single papers also highlighted the following barriers which may be relevant to clinical trials, including lack of follow-up during the recruitment process [32], lack of investigator/study team outreach to communities [28], challenging patient social and structural factors (e.g. homelessness) [24], difficulty locating eligible patients in clinic (HIV-related study) [24], and unavailability of research staff out-of-hours [35].

### Evidence-based strategies and enablers

The main evidence-based strategies and enablers to improving inclusion identified through the literature search are fully detailed in Additional File 1. The remainder of this section summarises the key evidence-based strategies identified within the literature.

#### Cultural competency training

There was a range of evidence suggesting that inclusion in trials was improved when staff had received specific training on that topic [13, 15, 19, 38–46]. This may include teaching study staff about cultural humility, existing health inequities, and the background and context as to how these have arisen [44], including previous research abuses (e.g. deception and mistreatment in research, such as the Tuskegee syphilis study). Acknowledgement of these was found to be important, particularly for African Americans [47]. It was also noted that applying knowledge of culturally important practices was also beneficial [16, 42, 48].

#### Community partnerships

A recurring theme was that strategies that closed the gap between the study team and the community were very effective [17, 19, 20, 38, 42, 46, 49–53], particularly community-based participatory research (CBPR) approaches [54, 55]. Some specific strategies around this included the use of a community advisory board [38, 46]; patient advocates/navigators, including to recruit participants [38, 49, 56]; ongoing partnerships with community members, leaders, groups, and organisations [19, 20, 38, 41, 42, 49, 50, 52, 56, 57]; direct outreach to community participants followed by electronic health record data for clinical information and follow-up [58]; oversight by a community panel [20]; and consultation with community members regarding study resources [49].

#### Personalised approach

Emphasis has been placed on strategies that lead to a more personal approach within clinical trials. These strategies may help people feel that they are seen both as an individual, as well as part of the groups with which they identify. Examples include building good rapport and relationships with participants [48, 51, 59, 60], individual communication styles [60], a human (i.e. not automated) phone call in the participant’s preferred language [61], birthday and holiday cards [57], thank you letters [53, 57], acknowledgement certificates [53], and relationship-centred recruitment and retention [43].

#### Multilingual materials and staff

A number of studies directly overcame language barriers for non-English speakers by providing bilingual staff [20, 42, 47, 52, 57, 59, 62], materials in non-English languages [20, 42, 49, 57, 62, 63], and/or an interpreter [42, 63]. Similarly, in a study conducted with deaf individuals, a variety of contact methods (video call and email) and materials in both sign language video and written English were found to help [17]. Many studies explicitly aimed to recruit study staff from under-served groups so that research teams are representative of the people being recruited, especially if from the local community [13, 17, 39, 47–50, 52], and some even matched study team members and participants on ethnicity [54].

#### Communication-specific strategies

Aside from language, a number of other strategies specifically related to communication have been implemented, including community providers or physicians sending letters of support to potential participants prior to study contact [42, 49, 62], mass mailing [64, 65], third party contact obtained at enrollment [59], out-of-hours contact [52, 59], keeping phone calls short [59], reminder calls or postcards [57, 59], regular study updates [57], maintaining up-to-date information [59], using social media for recruitment and retention (particularly Facebook) [56, 66], appropriate readability/simplified English materials [20, 44, 47], patient-centred/preferred communication methods [20], use of multimedia [15, 30], and appointment cancellations followed up vigorously [67].

#### Increasing understanding and trust

In order to tackle barriers related to a lack of understanding or trust in clinical trials, several studies provided educational sessions for communities [23, 39, 47, 49, 50, 65, 68], with one successfully employing teach/teach-back methodology [20]. Others aimed to build trust through communications [50] or by sharing patient safety information [47]. Using social proof was also a key strategy for improving trust either through participant testimonials [56, 63] or friends and family referrals [30, 64, 69].

#### Tackling logistical barriers

Multiple logistical barriers to recruitment and retention exist in clinical trials, and they remain a pertinent issue in under-served groups. Strategies that attempt to overcome these include flexible timings and locations of study visits [16, 20, 70], including home-based assessments [48]; providing childcare [42]; transport [24, 42]; and reducing costs associated with trial participation [42].

#### Additional strategies

Aside from these key strategies that were commonly referred to across the literature, some articles also highlighted the following enabling practices which may be relevant to clinical trials, including leadership from organisations and management around inclusion [38, 40, 42]; partnering with local healthcare centres/practices and clinical staff [20, 35, 43, 44, 49, 67, 71]; non-discriminatory inclusion/exclusion criteria [35, 48, 53, 64, 72]; recruitment targets for diverse groups [48, 52, 53, 64, 72]; electronic database to track participants throughout the study [44]; two-step method of collecting sex at birth and gender identity on data collection forms [46]; alleviating burdensome data collection [73]; study-branded items with study information, e.g. fridge magnets [53, 59]; and family involvement [42, 48].

## Discussion

### Summary of main findings

This rapid review aimed to synthesise the international published literature on studies that consider the specific barriers in relation to inclusion in clinical trials, and evidence of approaches that have been effective in overcoming these. It is notable that the majority of publications around equality, diversity, and inclusion (EDI) relate to ethnicity, whereas there is less contemporary literature around other protected characteristics. Furthermore, the majority of the literature is from the USA and focuses on cancer trials. The main barriers identified in the literature included language and communication, lack of trust, access to trials, eligibility criteria, attitudes and beliefs, lack of knowledge around clinical trials, and logistical and practical issues. The primary strategies to improve inclusion in clinical trials identified in the literature relate to staff cultural competency training, building community partnerships, taking a personalised approach, utilising multilingual materials and staff, communication-specific strategies, increasing understanding and trust, and tackling logistical barriers.

### Recommendations for policy and practice

There were two important points that arose from reviewing this literature. First, a multi-faceted approach is essential. The vast majority of studies found that multiple strategies were required to improve inclusion [13, 16, 17, 19, 20, 22, 24, 30, 35, 38–40, 42–45, 47–53, 56, 57, 59, 60, 62–64, 67, 72, 73]. Furthermore, most successful strategies had elements that operated at different levels, such as within the study team and within the community of interest.

Second, no single strategy was universally effective either within or between trials. For example, many studies found that providing incentives of some form was an enabler [16, 52, 57, 59, 67], whereas this was directly tested in one study that found no benefit of incentives [74]. Similarly, other studies found that strategies that worked well at one site did not necessarily work well at another site [52]. Effective strategies are also likely to be population-specific and may be contradictory. It is therefore important to consider the target population when choosing an approach. For example, a study of South Asians found that it was important to refer to gender throughout communications and have separate versions for men and women [42], whereas transgender individuals may have different preferences [46].

From reviewing the literature, we have made a series of 15 recommendations (Table 1) that may help improve inclusion within clinical trials and clinical research. The recommendations focus on 6 areas/strategies: [1] research staff covering the need for improving cultural competency and sensitivity of all clinical trial staff through training and ongoing learning, and the need to increase recruitment of staff from under-served groups [2]; communication including personalisation of communications, providing alternative languages, having video calling as an option, using social testimony, offering community outreach, and extending office hours [3]; establishing a diverse community advisory panel [4]; developing public education about clinical trials [5]; feasibility and or identification including examining demographics of excluded populations, encouraging the use of sites with high enrollment of under-served groups, exploring linkages with non-healthcare data sources, and creating a local registry of interested under-served groups; and [6] collecting participant data on both sex at birth and gender identity. Although much of the literature identified in this rapid review is drawn from the USA, our recommendations are general principles that are broadly applicable to similar clinical and healthcare contexts. Implementation of these recommendations may help improve representation of under-served groups in clinical trials which would lead to greater external validity of associated findings. Without external validity of research-informed treatments and services, delivering equitable high-quality care within healthcare systems is impaired.

**Table 1:** Recommendations for improving inclusion in clinical trials and clinical research

Type/area of strategy	Recommendation	Justification	Additional points to consider
Research staff	All research staff should receive cultural competency training	There was evidence that culturally sensitive study staff improved the recruitment and retention of diverse populations	Training should be given to staff at all levels so that cultural competency is present across research organisations. To foster an open environment where learning is continuous, it is also recommended that staff are encouraged to reflect on mistakes and lesson learnt [51]. Role-play may also be helpful as part of the training [44].
	Increase recruitment of staff from under-served groups	To better ensure that teams represent the communities that trials serve	This is also an important part of ensuring a culture that has inclusion at the forefront of its values.
Communication	Personalise communications as far as possible	There was strong evidence that people preferred to be seen as individuals	This could include, for example, having a consistent point of contact rather than having contact with lots of different individuals and sending personalised mailings such as birthday cards and thank you letters.
	Have video calling as a contact option	Some under-served groups require or prefer a visual option for communications, e.g. deaf individuals	This may also benefit other groups that rely on visual cues for communication.
	Alternative language options available for all communications	There was strong evidence that availability of non-English communications greatly assisted under-served groups	This relates to both written and verbal communications, and so bilingual staff or translators may be required. Also, consider non-foreign language options for other under-served groups, e.g. sign language and braille.
	Consider offering community outreach	Outreach to community leaders, groups, and providers was highly effective in several studies	Research teams could consider offering outreach (e.g. community events/meetings) to increase the participation of under-served groups.
	Include social proof where possible in communications	Social proof and validation were important enablers	Social proof could include, for example, testimonials. This may also help with building trust.
	Offer extended office hours	A lack of out-of-office availability was a consistent barrier	This could potentially cover evenings or weekends and could be delivered via telephone, email, or instant messenger chat. Under-served groups will be best placed to advise on how this could support them best.
Community	Establish a diverse community advisory panel for ongoing input into the research process	Closing the gap between researchers and under-served groups was consistently highlighted as an effective strategy	It is vital that the voices of under-served groups are heard. Building relationships with these groups and consulting regularly with them will help to ensure that existing inequities are not perpetuated.
Education	Include education about clinical trials for the public on study websites	A lack of knowledge about and trust in trials was a recurring barrier	This could focus particularly on the importance of inclusion in clinical trials and how the research organisation is working towards that.
Feasibility and or identification	Examine demographic summary of excluded populations	Eligibility criteria may directly or indirectly exclude under-served groups	Where possible (i.e. with access to local data), researchers should compare demographic summaries of the initial target population compared with that of eligible participants so that the generalisability of results can be determined.
	Encourage the use of sites with high enrollment of under-served groups	For research to be generalisable, it is key that the research goes where the need is and not only where investigators already are	This may require an initial understanding of which under-served groups are particularly relevant to the condition of interest.
	Explore whether linkages with non-healthcare data sources could increase trial access for people without access to healthcare	Inability to access healthcare and/or not being part of the traditional system were barriers to accessing trials	This could include, for example, working with refugee, prison, or homeless data providers.
	Consider creating a local registry of individuals from under-served groups who are interested in participating in trials	A diverse registry of people interested in participating in trials was an effective strategy	This could initially be piloted for one particular under-served group before being rolled out more widely.
Outcomes	Collect data on both sex at birth and gender identity	This was identified as an effective strategy among transgender individuals	This could also potentially benefit other under-served gender groups as well.

### Strengths and limitations

A key strength of this rapid review is the identification of a large evidence base on barriers and enablers to inclusion, which can be used to inform practice in clinical research. There are, however, limitations of the rapid review approach. By searching a single electronic database and only including peer-reviewed publications, potentially relevant publications may have been omitted, therefore, introducing some bias. Examples of studies that would be systematically excluded from the search are grey literature, as well as studies that include barriers or enablers as secondary aspects that are not clearly searchable within the title or abstract. However, it is unlikely that the barriers or enablers in such studies would be systematically different from those identified in this search, and so the results are presumed to be unbiased. In addition, the use of a single reviewer to ensure consistency and reduce the time required for this review may have also introduced bias. A further limitation is the lack of a quality assessment of included studies. By not appraising the methodological quality of studies, the quality of the evidence on which our findings are based is not known.

## Conclusion

This review identified a range of barriers relating to inclusion, and the available literature suggests these issues may manifest differently depending on the population. A number of strategies to overcome these barriers to inclusion were identified, but the implementation of multiple approaches and at differing levels may be required. Based on the available evidence, we made a series of recommendations that, if implemented, may help improve inclusion within clinical trials and clinical research.

## Supplementary Information

**Additional file 1.** EDI Rapid Review Paper Author Responses

## Data Availability

The datasets used and/or analysed during the current study are available from the corresponding author on reasonable request.

## References

[1] Treweek S, Forouhi NG, Narayan KMV, Khunti K (2020). COVID-19 and ethnicity: who will research results apply to?. Lancet..

[2] NIHR. Improving inclusion of under-served groups in clinical research: guidance from INCLUDE project 2020 [August 2020 v1.0:[Available from: https://www.nihr.ac.uk/documents/improving-inclusion-of-under-served-groups-in-clinical-research-guidance-from-include-project/25435.

[3] Mason S, Hussain-Gambles M, Leese B, Atkin K, Brown J (2003). Representation of South Asian people in randomised clinical trials: analysis of trials’ data. BMJ..

[4] Bartlett C, Davey P, Dieppe P, Doyal L, Ebrahim S, Egger M (2003). Women, older persons, and ethnic minorities: factors associated with their inclusion in randomised trials of statins 1990 to 2001. Heart.

[5] Heiat A, Gross CP, Krumholz HM (2002). Representation of the elderly, women, and minorities in heart failure clinical trials. Arch Intern Med.

[6] Hall WD (1999). Representation of blacks, women, and the very elderly (aged > or = 80) in 28 major randomized clinical trials. Ethn Dis.

[7] Gardiner T, Cooke G, Fidler S, Cooper N, Young L (2020). The under-representation of BAME patients in the COVID-19 Recovery trial at a major London NHS Trust. J Infect.

[8] Dawson S, Campbell SM, Giles SJ, Morris RL, Cheraghi-Sohi S (2018). Black and minority ethnic group involvement in health and social care research: a systematic review. Health Expect.

[9] Iqbal H, West J, Haith-Cooper M, McEachan RRC (2021). A systematic review to identify research priority setting in Black and minority ethnic health and evaluate their processes. PLOS ONE.

[10] Pan D, Sze S, Minhas JS, Bangash MN, Pareek N, Divall P, Williams CML, Oggioni MR, Squire IB, Nellums LB, Hanif W, Khunti K, Pareek M (2020). The impact of ethnicity on clinical outcomes in COVID-19: a systematic review. EClinicalMedicine.

[11] Witham MD, Anderson E, Carroll C, Dark PM, Down K, Hall AS, Knee J, Maier RH, Mountain GA, Nestor G, Oliva L, Prowse SR, Tortice A, Wason J, Rochester L, On behalf of the INCLUDE writing group (2020). Developing a roadmap to improve trial delivery for under-served groups: results from a UK multi-stakeholder process. Trials.

[12] Harrison R, Walton M, Chitkara U, Manias E, Chauhan A, Latanik M, Leone D (2020). Beyond translation: engaging with culturally and linguistically diverse consumers. Health Expect.

[13] Bass SB, D'Avanzo P, Alhajji M, Ventriglia N, Trainor A, Maurer L, Eisenberg R, Martinez O (2020). Exploring the engagement of racial and ethnic minorities in HIV treatment and vaccine clinical trials: a scoping review of literature and implications for future research. AIDS Patient Care STDS.

[14] Byrne MM, Tannenbaum SL, Glück S, Hurley J, Antoni M (2014). Participation in cancer clinical trials: why are patients not participating?. Med Decis Making.

[15] Hughson JA, Woodward-Kron R, Parker A, Hajek J, Bresin A, Knoch U, Phan T, Story D (2016). A review of approaches to improve participation of culturally and linguistically diverse populations in clinical trials. Trials.

[16] Rooney LK, Bhopal R, Halani L, Levy ML, Partridge MR, Netuveli G, Car J, Griffiths C, Atkinson J, Lindsay G, Sheikh A (2011). Promoting recruitment of minority ethnic groups into research: qualitative study exploring the views of South Asian people with asthma. J Public Health (Oxf).

[17] Anderson ML, Wolf Craig KS, Ziedonis DM (2017). Barriers and facilitators to deaf trauma survivors' help-seeking behavior: lessons for behavioral clinical trials research. J Deaf Stud Deaf Educ.

[18] Isaacs T, Murdoch J, Demjén Z, Stevenson F. Examining the language demands of informed consent documents in patient recruitment to cancer trials using tools from corpus and computational linguistics. Health (London). 2020:1363459320963431. 10.1177/1363459320963431 PMC9163777 33045861

[19] Boden-Albala B, Carman H, Southwick L, Parikh NS, Roberts E, Waddy S, Edwards D (2015). Examining barriers and practices to recruitment and retention in stroke clinical trials. Stroke.

[20] Fischer SM, Kline DM, Min SJ, Okuyama S, Fink RM (2017). Apoyo con Cariño: strategies to promote recruiting, enrolling, and retaining Latinos in a cancer clinical trial. J Natl Compr Canc Netw.

[21] Le Rouzic MA, Claudot F (2020). Characteristics of parental decision-making for children with advanced cancer who are offered enrollment in early-phase clinical trials: a systematic review. Pediatr Hematol Oncol.

[22] Clark LT, Watkins L, Piña IL, Elmer M, Akinboboye O, Gorham M, Jamerson B, McCullough C, Pierre C, Polis AB, Puckrein G, Regnante JM (2019). Increasing diversity in clinical trials: overcoming critical barriers. Curr Probl Cardiol.

[23] Cunningham-Erves J, Barajas C, Mayo-Gamble TL, McAfee CR, Hull PC, Sanderson M (2020). Formative research to design a culturally-appropriate cancer clinical trial education program to increase participation of African American and Latino communities. BMC Public Health.

[24] Hoffman KA, Baker R, Kunkel LE, Waddell EN, Lum PJ, McCarty D, Korthuis PT (2019). Barriers and facilitators to recruitment and enrollment of HIV-infected individuals with opioid use disorder in a clinical trial. BMC Health Serv Res.

[25] Lee GE, Ow M, Lie D, Dent R (2016). Barriers and facilitators for clinical trial participation among diverse Asian patients with breast cancer: a qualitative study. BMC Womens Health.

[26] Bloch F, Charasz N (2014). Attitudes of older adults to their participation in clinical trials: a pilot study. Drugs Aging.

[27] Javid SH, Unger JM, Gralow JR, Moinpour CM, Wozniak AJ, Goodwin JW, Lara PN, Williams PA, Hutchins LF, Gotay CC, Albain KS (2012). A prospective analysis of the influence of older age on physician and patient decision-making when considering enrollment in breast cancer clinical trials (SWOG S0316). Oncologist.

[28] Frew PM, Saint-Victor DS, Isaacs MB, Kim S, Swamy GK, Sheffield JS, et al. Recruitment and retention of pregnant women into clinical research trials: an overview of challenges, facilitators, and best practices. Clin Infect Dis. 2014;59 Suppl 7(Suppl 7):S400-7. 10.1093/cid/ciu726 PMC4303058 25425718

[29] Barrett NJ, Rodriguez EM, Iachan R, Hyslop T, Ingraham KL, Le GM (2020). Factors associated with biomedical research participation within community-based samples across 3 National Cancer Institute-designated cancer centers. Cancer.

[30] Rivers D, August EM, Sehovic I, Lee Green B, Quinn GP (2013). A systematic review of the factors influencing African Americans’ participation in cancer clinical trials. Contemp Clin Trials.

[31] Canouï-Poitrine F, Lièvre A, Dayde F, Lopez-Trabada-Ataz D, Baumgaertner I, Dubreuil O, Brunetti F, Coriat R, Maley K, Pernot S, Tournigand C, Hagege M, Aparicio T, Paillaud E, Bastuji-Garin S (2019). Inclusion of older patients with cancer in clinical trials: the SAGE prospective multicenter cohort survey. Oncologist.

[32] Caldieraro-Bentley AJ, Kelechi TJ, Treat-Jacobson D, Mueller M (2018). Challenges in recruitment of persons with peripheral artery disease for exercise studies. J Vasc Nurs.

[33] Shepherd V, Wood F, Griffith R, Sheehan M, Hood K. Protection by exclusion? The (lack of) inclusion of adults who lack capacity to consent to research in clinical trials in the UK. Trials. 202019. p. 474. 10.1186/s13063-019-3603-1 PMC6683336 31382999

[34] Webb Hooper M, Asfar T, Unrod M, Dorsey A, Correa JB, Brandon KO, Simmons VN, Antoni MA, Koru-Sengul T, Lee DJ, Brandon TH (2019). Reasons for exclusion from a smoking cessation trial: an analysis by race/ethnicity. Ethn Dis.

[35] Kaur G, Smyth RL, Powell CV, Williamson P (2016). A survey of facilitators and barriers to recruitment to the MAGNETIC trial. Trials.

[36] Rivera-Goba MV, Dominguez DC, Stoll P, Grady C, Ramos C, Mican JM (2011). Exploring decision-making of HIV-infected Hispanics and African Americans participating in clinical trials. J Assoc Nurses AIDS Care.

[37] Alahmad G (2018). Informed consent in pediatric oncology: a systematic review of qualitative literature. Cancer Control.

[38] Brooks SE, Muller CY, Robinson W, Walker EM, Yeager K, Cook ED, Friedman S, Somkin CP, Brown CL, McCaskill-Stevens W (2015). Increasing minority enrollment onto clinical trials: practical strategies and challenges emerge from the NRG oncology accrual workshop. J Oncol Pract.

[39] Ezeugwu CO, Laird A, Mullins CD, Saluja DS, Winston RA (2011). Lessons learned from community-based minority health care serving system participation in an NIH clinical trial. J Natl Med Assoc.

[40] Regnante JM, Richie NA, Fashoyin-Aje L, Vichnin M, Ford M, Roy UB, Turner K, Hall LL, Gonzalez E, Esnaola N, Clark LT, Adams HC, Alese OB, Gogineni K, McNeill L, Petereit D, Sargeant I, Dang J, Obasaju C, Highsmith Q, Lee SC, Hoover SC, Williams EL, Chen MS (2019). US cancer centers of excellence strategies for increased inclusion of racial and ethnic minorities in clinical trials. J Oncol Pract.

[41] Wallington SF, Dash C, Sheppard VB, Goode TD, Oppong BA, Dodson EE (2016). Enrolling minority and underserved populations in cancer clinical research. Am J Prev Med.

[42] Waheed W, Husain N, Allen G, Atif N, Aseem S, Waquas A, Garrett C, Sheikh S, Syed A, Gask L, Bower P (2015). Recruitment strategies for British South Asians in 5 depression trials: a mixed method study. J Affect Disord.

[43] Horowitz CR, Sabin T, Ramos M, Richardson LD, Hauser D, Robinson M, Fei K (2019). Successful recruitment and retention of diverse participants in a genomics clinical trial: a good invitation to a great party. Genet Med.

[44] Goff SL, Youssef Y, Pekow PS, White KO, Guhn-Knight H, Lagu T, Mazor KM, Lindenauer PK (2016). Successful strategies for practice-based recruitment of racial and ethnic minority pregnant women in a randomized controlled trial: the IDEAS for a Healthy Baby Study. J Racial Ethn Health Disparities.

[45] Herrera AP, Snipes SA, King DW, Torres-Vigil I, Goldberg DS, Weinberg AD. Disparate inclusion of older adults in clinical trials: priorities and opportunities for policy and practice change. Am J Public Health. 2010;100 Suppl 1(Suppl 1):S105-12. 10.2105/AJPH.2009.162982 PMC2837461 20147682

[46] Siskind RL, Andrasik M, Karuna ST, Broder GB, Collins C, Liu A, et al. Engaging transgender people in NIH-funded HIV/AIDS clinical trials research. J Acquir Immune Defic Syndr. 2016;72 Suppl 3(Suppl 3):S243-7. 10.1097/QAI.0000000000001085 PMC4969066 27429190

[47] Ford ME, Siminoff LA, Pickelsimer E, Mainous AG, Smith DW, Diaz VA, Soderstrom LH, Jefferson MS, Tilley BC (2013). Unequal burden of disease, unequal participation in clinical trials: solutions from African American and Latino community members. Health Soc Work.

[48] Selak V, Crengle S, Elley CR, Wadham A, Harwood M, Rafter N, Bullen C, Pillai A, Arroll B, Rodgers A Recruiting equal numbers of indigenous and non-indigenous participants to a ‛polypill’ randomized trial Int J Equity Health 2013;12:44, 1, DOI: 10.1186/1475-9276-12-44. 10.1186/1475-9276-12-44 PMC3694525 23800177

[49] Heller C, Balls-Berry JE, Nery JD, Erwin PJ, Littleton D, Kim M, Kuo WP (2014). Strategies addressing barriers to clinical trial enrollment of underrepresented populations: a systematic review. Contemp Clin Trials.

[50] Coakley M, Fadiran EO, Parrish LJ, Griffith RA, Weiss E, Carter C (2012). Dialogues on diversifying clinical trials: successful strategies for engaging women and minorities in clinical trials. J Womens Health (Larchmt).

[51] Maar MA, Beaudin V, Yeates K, Boesch L, Liu P, Madjedi K, Perkins N, Hua-Stewart D, Beaudin F, Wabano MJ, Tobe SW (2019). Wise practices for cultural safety in electronic health research and clinical trials with indigenous people: secondary analysis of a randomized clinical trial. J Med Internet Res.

[52] Duda C, Mahon I, Chen MH, Snyder B, Barr R, Chiles C, Falk R, Fishman EK, Gemmel D, Goldin JG, Brown K, Munden RF, Vydareny K, Aberle DR (2011). Impact and costs of targeted recruitment of minorities to the National Lung Screening Trial. Clin Trials.

[53] Falcon R, Bridge DA, Currier J, Squires K, Hagins D, Schaible D, Ryan R, Mrus J, GRACE Study Group. Recruitment and retention of diverse populations in antiretroviral clinical trials: practical applications from the gender, race and clinical experience study. J Womens Health (Larchmt) 2011;20(7):1043-1050, DOI: 10.1089/jwh.2010.2504. 10.1089/jwh.2010.2504 PMC3130514 21663416

[54] Burlew AK, Weekes JC, Montgomery L, Feaster DJ, Robbins MS, Rosa CL (2011). Conducting research with racial/ethnic minorities: methodological lessons from the NIDA Clinical Trials Network. Am J Drug Alcohol Abuse..

[55] De las Nueces D, Hacker K, DiGirolamo A, Hicks LS (2012). A systematic review of community-based participatory research to enhance clinical trials in racial and ethnic minority groups. Health Serv Res.

[56] Adams SA, Heiney SP, Brandt HM, Wirth MD, Khan S, Johnson H, Davis L, Wineglass CM, Warren-Jones TY, Felder TM, Drayton RF, Davis B, Farr DE, Hébert JR (2015). A comparison of a centralized versus de-centralized recruitment schema in two community-based participatory research studies for cancer prevention. J Community Health.

[57] Sacheck JM, Van Rompay MI, Olson EM, Chomitz VR, Goodman E, Gordon CM (2015). Recruitment and retention of urban schoolchildren into a randomized double-blind vitamin D supplementation trial. Clin Trials.

[58] Zimmerman LP, Goel S, Sathar S, Gladfelter CE, Onate A, Kane LL, Sital S, Phua J, Davis P, Margellos-Anast H, Meltzer DO, Polonsky TS, Shah RC, Trick WE, Ahmad FS, Kho AN (2018). A novel patient recruitment strategy: patient selection directly from the community through linkage to clinical data. Appl Clin Inform.

[59] Barnett J, Aguilar S, Brittner M, Bonuck K (2012). Recruiting and retaining low-income, multi-ethnic women into randomized controlled trials: successful strategies and staffing. Contemp Clin Trials.

[60] Lavender V, Gibson F, Brownsdon A, Fern L, Whelan J, Pearce S (2019). Health professional perceptions of communicating with adolescents and young adults about bone cancer clinical trial participation. Support Care Cancer.

[61] Andreae MH, Nair S, Gabry JS, Goodrich B, Hall C, Shaparin N (2017). A pragmatic trial to improve adherence with scheduled appointments in an inner-city pain clinic by human phone calls in the patient’s preferred language. J Clin Anesth.

[62] Wang JH, Sheppard VB, Liang W, Ma GX, Maxwell AE (2014). Recruiting Chinese Americans into cancer screening intervention trials: strategies and outcomes. Clin Trials.

[63] Napoles A, Cook E, Ginossar T, Knight KD, Ford ME (2017). Applying a conceptual framework to maximize the participation of diverse populations in cancer clinical trials. Adv Cancer Res.

[64] Ramsey TM, Snyder JK, Lovato LC, Roumie CL, Glasser SP, Cosgrove NM, Olney CM, Tang RH, Johnson KC, Still CH, Gren LH, Childs JC, Crago OL, Summerson JH, Walsh SM, Perdue LH, Bankowski DM, Goff DC, for the SPRINT Study Research Group (2016). Recruitment strategies and challenges in a large intervention trial: Systolic Blood Pressure Intervention Trial. Clin Trials.

[65] Sturgeon KM, Hackley R, Fornash A, Dean LT, Laudermilk M, Brown JC, Sarwer DB, DeMichele AM, Troxel AB, Schmitz KH (2018). Strategic recruitment of an ethnically diverse cohort of overweight survivors of breast cancer with lymphedema. Cancer.

[66] Wisk LE, Nelson EB, Magane KM, Weitzman ER (2019). Clinical trial recruitment and retention of college students with type 1 diabetes via social media: an implementation case study. J Diabetes Sci Technol.

[67] Vander Stoep A, Myers K (2013). Methodology for conducting the children’s attention-deficit hyperactivity disorder telemental health treatment study in multiple underserved communities. Clin Trials.

[68] Amorrortu RP, Arevalo M, Vernon SW, Mainous AG, Diaz V, McKee MD (2018). Recruitment of racial and ethnic minorities to clinical trials conducted within specialty clinics: an intervention mapping approach. Trials.

[69] Koziol-McLain J, McLean C, Rohan M, Sisk R, Dobbs T, Nada-Raja S, Wilson D, Vandal AC (2016). Participant recruitment and engagement in automated eHealth trial registration: challenges and opportunities for recruiting women who experience violence. J Med Internet Res..

[70] Smorenburg AJ, Oosterman BJ, Grobbee DE, Bonten MJ, Roes KC (2014). Effects of recruitment strategies and demographic factors on inclusion in a large scale vaccination trial in adults 65 years and older. Vaccine.

[71] Tilley BC, Mainous AG, Elm JJ, Pickelsimer E, Soderstrom LH, Ford ME (2012). A randomized recruitment intervention trial in Parkinson’s disease to increase participant diversity: early stopping for lack of efficacy. Clin Trials.

[72] Freedman RA, Dockter TJ, Lafky JM, Hurria A, Muss HJ, Cohen HJ, Jatoi A, Kemeny MM, Ruddy KJ (2018). Promoting accrual of older patients with cancer to clinical trials: an alliance for clinical trials in oncology member survey (A171602). Oncologist.

[73] Yu SH, Gumport NB, Mirzadegan IA, Mei YJ, Hein K, Harvey AG (2020). Addressing the challenges of recruitment and retention in sleep and circadian clinical trials. Behav Sleep Med.

[74] Jennings CG, MacDonald TM, Wei L, Brown MJ, McConnachie L, Mackenzie IS (2015). Does offering an incentive payment improve recruitment to clinical trials and increase the proportion of socially deprived and elderly participants?. Trials.

