# Supplementary material for: Promoting inclusion in clinical trials—a rapid review of the literature and recommendations for action
Source: Trials. 2021 Dec 4;22:880. doi: 10.1186/s13063-021-05849-7 (PMC8643184; doi:10.1186/s13063-021-05849-7)
Supplement: Supplementary file 1 — Additional file 1. EDI Rapid Review Paper Author Responses [file 13063_2021_5849_MOESM1_ESM.docx]

**Supplementary Table 1. Review Search Strategies**

(underserved OR under-served OR underrepresented OR under-represented OR seldom-heard OR “seldom heard” OR “hard to reach” OR “hard-to-reach” OR minority OR minorities OR marginalised OR marginalized OR discriminated OR disability OR “mental incapacity” OR ethnicity OR gender OR migrant* OR “asylum seeker*” OR traveller* OR deprived OR race OR age OR “socio-economic status” OR “care home*” OR prison OR homeless)

AND

(challenge* OR barrier* OR facilitator* OR approach* OR strategy OR strategies)

AND

(diversity OR inclusion OR equality)

AND

“clinical trial*”

# **Supplementary Table 2. Strategies identified as improving inclusion in clinical trials**

| **First author** | **Year** | **Country** | **Study type** | **N** | **Underserved**  **group(s)** | **Conditions** | **Key strategies/enablers** |
| --- | --- | --- | --- | --- | --- | --- | --- |
|  |  |  |  |  |  |  |  |
|  |  |  |  |  |  |  |  |
| **General/multiple** | | | | | | | |
| Zimmerman(1) | 2018 | USA | Case study | 1 trial; individuals NR | General | Aspirin dosing | - Direct outreach to community participants then electronic health record data for clinical information and follow-up |
| Brooks(2) | 2015 | USA | Workshop | NR | General | Cancer | - Cooperative groups  - Large health care system  - Culturally sensitive investigators and coordinators  - Community advisory board  - Partnership with local churches, community groups, and language-based media outlets on an ongoing basis  - Patient advocate  - Institutional leadership |
| Ezeugwu(3) | 2011 | USA | Qualitative | NR | General | General | - Cultural competence and sensitivity  - Minority staff  - Clarity of study purpose and aims at outset  - Reinforcement with educational sessions |
| Heller(4) | 2014 | USA | Systematic review | 20 trials | General | General | - Partnerships with community members, leaders and organisations  - Outreach to providers  - Community research staff  - Use of community members to recruit participants  - Training/education around clinical trials  - Culturally/linguistically sensitive materials and direct outreach  - Community providers sent letters of support  - Recruiting at local/ community practices  - Consultation with patients, providers and staff regarding materials  - Pilot testing recruitment approaches  - Regular interactions between study team and community providers  - Research staff from community  - Research staff recruit at community practices  - Patient navigators  - Provided infrastructure at practices  - Incentives |
| Coakley(5) | 2012 | USA | Conference | NR | Women and ethnic minorities | General | - Recruit female and ethnic minority physicians  - Build trust through communication  - Educate to raise awareness  - Involve communities |
| Yu(6) | 2020 | USA | Case studies | 2 trials; 297 individuals | Adolescents; low income ethnic minorities | Sleep | - Community based recruitment  - Reducing stigma  - Alleviating burdensome data collection |
| Boden-Albala(7) | 2015 | USA | Mixed-methods | 93 researchers | General | Stroke | - Standardized project management procedures and protocols (e.g. travel re-imbursement)  - Research staff cultural competency and communication training  - Community partnerships to guide research process |
| Ramsey(8) | 2016 | USA | Case study | 1 RCT; 9361 participants | Older age (≥75 years), women or ethnic minorities | Systolic blood pressure | - Targets for minority groups  - Mass mailing  - Referrals  - Regular monitoring of recruitment targets in subgroups  - Adding new clinics as needed  - Modifying eligibility criteria if recruitment lags |
|  |  |  |  |  |  |  |  |
| **Ethnic minority groups** | | | | | | | |
| Romero(9) | 2014 | USA | Case study | 1 trial; 2311 individuals | African Americans | Alzheimer’s disease | - Creation of a diverse registry of people interested in participating in clinical trials |
| Rooney(10) | 2011 | UK | Qualitative study | 58 | South Asians | Asthma | - Flexible timing and locations  - Respect of cultural and religious sensitivities around gender segregation  - Incentives |
| Barnett(11) | 2012 | USA | Case studies | 2 RCTs; 907 individuals | Multi-ethnic low-income women | Breastfeeding | - Gift card incentives  - 3^rd^ party contact obtained at enrollment  - Maintaining up-to-date information  - High availability of bilingual staff  - Out-of-hours contact  - Business cards and magnets with study information  - Reminder postcards  - Good rapport with participants  - Short phone calls |
| Adams(12) | 2015 | USA | Case studies | 2 RCTs; 672 individuals | African Americans | Cancer prevention | - Community-based participatory recruitment either de-centralized (via local churches) or centralized (via single lay community individual)  - Participant testimonials  - Updating instructions  - Reinforcement of program details  - Community social network on Facebook  *Factors that did not improve diversity:*  *- Different staff members making data collection calls*  *- Health fairs*  *- Lengthy questionnaires* |
| Diehl(13) | 2011 | USA | Review | 10 trials | African Americans, Hispanics/Latino Americans | Cancer | - Study design (disease stage and type of therapy) |
| Rivers(14) | 2013 | USA | Systematic Review | 31 trials | African Americans | Cancer | - Recommendations from physicians, family, and friends  - Multimedia  - Culturally-appropriate recruitment |
| Wang(15) | 2014 | USA | Case studies | 2 trials; 1119 individuals | First-generation Asian American immigrants | Cancer | - In-person community fairs (e.g. Chinese schools, stores, health fairs, personal networks)  - Bilingual staff  - Linguistically appropriate fliers and brochures  - Physician letter followed by telephone calls |
| Napoles(16) | 2017 | USA | Case studies | 3 trials | Ethnic minorities | Cancer | - Culturally and linguistically appropriate outreach, education and research studies that were accessible in local communities  - Infrastructure to support engagement of key stakeholders serving minority communities  - Testimonials from ethnically diverse cancer survivors  - Availability of medical interpretation services  - Infrastructure that facilitated engagement of clinicians who engage in care for minority populations |
| Fischer(17) | 2017 | USA | Case study | 1 RCT; 223 individuals | Latino | Cancer | - Community input and liaison  - Oversight by community panel  - Translated materials  - Bilingual research personnel  - Materials written at 5^th^/6^th^ grade reading level  - Teach/teach-back methods  - Patient-centred/preferred communication methods  - Relationships with community cancer clinics  - Educational and financial compensation to sites  - Flexible timing and locations for visits |
| Sturgeon(18) | 2018 | USA | Case study | 1 trial; 2295 individuals | Black women | Cancer | - Community education sessions  - Mailings |
| Nickell(19) | 2019 | USA | RCT | 133 | Ethnically-diverse low-income women | Cancer | *Factors that did not improve diversity:*  *- Patient navigator intervention* |
| Tabriz(20) | 2019 | USA | Case study | 1 trial; 4518 individuals | Black or Asian individuals | Cancer | *Factors that did not improve diversity:*  *- Online portal* |
| Regnante(21) | 2019 | USA | Qualitative | 14 | Ethnic minorities | Cancer | - Commitment and centre leadership  - Investigator training and mentoring  - Community engagement  - Patient engagement  - Operational practices  - Increased engagement of HCPs  - Formal processes for obtaining patient/caregiver input on research projects  - Allocation of resources to improve health disparities  - Dedication of research staff to ethnic minority engagement |
| Cunningham-Erves(22) | 2020 | USA | Qualitative | NR | African American, Latino | Cancer | - Education programme about clinical trials |
| Wallington(23) | 2016 | USA | Cultural competency frameworks | 6 trials, 559 individuals | Low-income African American women | Cancer, smoking cessation and HPV | - Capacity to acquire cultural knowledge about patients  - Embedding programmes and research in the community |
| Selak(24) | 2013 | Australia | Case study | 1 trial; 513 individuals | Indigenous people (Maori) | Cardiovascular disease | - Maori researchers and nurses  - Recruitment target  - Home-based assessment  - Establishing and continuing nurse-participant relationships  - Family involvement prior to enrollment  - Acknowledgement of culturally important procedures, interactions, languages and manners  - Oversampling of potentially eligible Maori patients  - Targeted practices with high Maori enrollment  - Lower screening thresholds for Maori  - Continued Maori recruitment after non-Maori recruitment had ended |
| Waheed(25) | 2015 | UK | Case studies | 5 trials; 292 individuals | South Asians | Depression, post-natal depression, DM or IHD | - Changing exploratory models of illness  - Developing community engagement  - Working with the media  - Collaborating with key persons  - Patient and public involvement  - Fostering ongoing academic-community partnerships  - Researcher training  - Providing psycho-education  - Getting senior investigators involved  - Linguistically matched staff  - Use of interpreters  - Native language written materials  - Working with places of worship  - Demonstrate religious knowledge and sensitivity  - Reassuring the participants  - Contacting participants through the GP  - Sensitivity to gender needs  - Referring to gender in written communication  - Logistic needs  - Getting family participation  - Providing childcare  - Reassuring participants around immigration  - Transport availaibility  - Selecting local venues  - Meeting extra costs  - Selection of incentives  - Offering food and tea  - Accomodating special requests  - Options and choice around locations and timings  - Working with husbands and the family  - Recruiting from multiple sources  - Employing culturally aware staff (including willingness to share some personal information and dress modestly)  - Collaborative working  - Raising awareness of unmet needs of ethnic minorities  - Inviting ethnic minorities to be co-investigators  - Culturally appropriate assessments  - Select ethnically dense geographical areas  - Use non-health recruitment sources |
| Horowitz(26) | 2019 | USA | Case study | 1 RCT; 2698 individuals | African-Americans | Genetic testing | - Stakeholder engagement  - Formative work  - Clinician buy-in  - Study materials and stipends  - Recruiter training and oversight  - Efficient patient identification in a variety of settings  - Flexible, targeted outreach and scheduling  - Study branding  - Relationship-centred recruitment and retention  - Collect data for impact of strategies |
| Bass(27) | 2020 | USA | Literature review | 31 studies | Ethnic minorities | HIV treatment and vaccine | - Training HCPs on importance of recruiting diverse populations  - Creating interdisciplinary research teams that better represent who is being recruited  - Culturally competent trial designs |
| Maar(28) | 2019 | Canada | Qualitative | 142 | Indigenous people | Hypertension | - Building and maintaining a respectful relationship  - Good communication and support for the local team  - Commitment to co-designing the innovation  - Supporting task shifting with the local team  - Reflecting on mistakes and lessons learnt |
| Duda(29) | 2011 | USA | Case study | 1 RCT; 18,842 individuals | Ethnic minorities | Lung screening | - A priori targets  - Dedicated financial resources  - Working with community-based leaders and groups  - Ethnically-diverse, bilingual staff  - Infrastructure support to disseminate communication  - Media advertisements  - Incentives  - Out-of-hours contact |
| Andreae(30) | 2017 | USA | RCT | 963 | Mostly African-American and Hispanic | Pain | - Human phone call in patient’s preferred language |
| Tilley(31) | 2012 | USA | Mixed-methods ancillary trial | 46 sites | Ethnic minorities | Parkinson’s Disease | - Use of existing physician relationships  - Untargeted community outreach  - Extensive efforts to overcome participants’ barriers  *- Recruitment intervention stopped early for lack of efficacy* |
| Goff(32) | 2016 | USA | Case study | 1 RCT; 746 individuals | Ethnic minority women | Pregnancy | - Teaching study staff about health inequities  - Role-playing skills to develop rapport and trust  - Partnering with clinical staff  - Use of electronic systems for pre-screening and determining when eligible people were in clinic  - Electronic database to track participants through study  - Flexible protocol  - Stipend  - Consideration of literacy levels |
| Brown(33) | 2015 | USA | RCT | 445 | African American, Asian, Pacific Islander, Latina, and/or non-English preferred language | Pregnancy with gestational diabetes or impaired glucose tolerance | *Found not to affect diversity: Targeted recruitment letters acknowledging health disparities and risks specific to recipient’s ethnic group* |
| Burlew(34) | 2011 | USA | Case studies | 25 RCTs | Ethnic minorities | Substance abuse | - Community-based participatory research approach  - Matching participant and study team member on ethnicity  - Assessing measurement equivalence |
| Hughson(35) | 2016 | Australia | Review | NR | Culturally and linguistically diverse | General | - Culturally and linguistically sensitive multimedia tools  - Researcher education  - Incorporate multimedia resources into informed consent process |
| Amorrortu(36) | 2018 | USA | RCT | 50 clinics | Ethnic minorities | General | - 5 educational modules, 1 in-person and 4 web-based, plus technical assistance calls to coordinators |
| Clark(37) | 2019 | USA | Mixed-methods | 66 | African-American, Hispanic, Asian | General | - Building trust  - Common understanding of the goal  - Clinical trial awareness  - Optimizing the role of the study co-ordinator  - Addressing resource and time constraints |
| De Las Nueces(38) | 2012 | USA | Systematic review | 19 papers | Ethnic minorities | General | - Community-based participatory research |
| Ford(39) | 2013 | USA | Qualitative | 32 | African-American, Latino | General | - Addressing costs  - Recruiting in community contexts  - Conducting community and individualized patient education  - Sharing patient safety information  - Diversifying research teams  - Recognising past research abuses  - Increasing community trust  - Low-literacy materials  - Bilingual clinicians and advocates  - Immigration status neither documented nor prevent participation |
| Yamal(40) | 2014 | USA | Case study | 1 trial; 289 individuals | Ethnic minorities | Traumatic brain injury | *Factors that did not improve diversity:*  *- Availability of exception from informed consent* |
|  |  |  |  |  |  |  |  |
| **Children** | | | | | | | |
| Vander Stoep(41) | 2013 | USA | Case study | 1 trial; 223 individuals | Children in nonmetropolitan communities | ADHD | - Widened participation to healthcare providers without previous involvement in the research  - Pre-trial visits to providers with information packs, flyers and quarterly newsletters  - PI sent a treatment option letter for referred children who did not meet eligibility criteria  - Clinic note sent to usual HCP after each trial session and smooth referral back at end of trial  - Patient-centred care and therapeutic alliance  - Cancellations followed up vigorously  - Incentives |
| Kaur(42) | 2016 | UK | Survey | 206 | Children | Asthma | - Motivation and experience of the study teams  - Effective communication and coordination between teams at site and between sites and the Clinical Trials Unit  - Presence of dedicated research nurses  - Good trial management  - Clinical trial publicity  - Simple inclusion criteria  - Effective communication  - Presentation of trial information in a simple and clear manner |
| Le Rouzic(43) | 2020 | France | Systematic review | 30 trials | Children | Cancer | - Therapeutic alliance  - Empathetic attitude  - Timely delivery of accurate information  - Due weight to the voice of the child  - Age-adapted information |
| Sacheck(44) | 2015 | USA | Case study | 1 trial; 691 individuals | Urban schoolchildren | Vitamin D supplementation | - School/district/PTA meetings  - Regular study updates  - Incentives  - Enlisting school staff support  - Open houses  - Classroom presentations to children  - Materials in 5 languages  - Monthly telephone reminders and troubleshooting  - Wristbands etc with study logo  - Retention letters  - Bilingual callers  - Birthday and Valentine’s Day cards  - Thank you letters |
|  |  |  |  |  |  |  |  |
| **Adolescents and/or young adults** | | | | | | | |
| Fern(45) | 2014 | UK | Mixed-methods | 49 RCTs | Teenagers and young adults | Cancer | *Inconclusive evidence:*  *- Age inclusion criteria*  *- Availability of trials*  *- Access to and awareness of trials*  *- Acceptability* |
| Lavender(46) | 2019 | UK | Qualitative | 18 | Adolescents and young adults | Cancer | - Credibility through team expertise  - Developing specialist communication skills through reflection on practice  - Inclusive approaches to education and training  - Individual communication styles  - Patient-centred approach  - Creating time to form trusting relationships  - Effective team working |
| Knopf(47) | 2017 | USA | Mixed-methods | 58 | Adolescents and young adults | HIV prevention | - Feeling well-informed  - Supported by study staff  - Support with decision-making |
| Wisk(48) | 2019 | USA | Case study | 1 trial; 227 individuals | College students | Type 1 diabetes | - Social media particularly Facebook |
|  |  |  |  |  |  |  |  |
| **Older adults** | | | | | | | |
| Freedman(49) | 2018 | USA | Survey | 1146 | Older adults (>65) | Cancer | - More dedicated trials for older participants  - Minimizing exclusion criteria focused on comorbidity  - Developing independent strategies for those aged ≥65 years and for those aged ≥70 years  - Specific targets |
| Canoui-Poitrine(50) | 2019 | France | Cohort study | 37 RCTs; 577 individuals | Older adults (>65 years) | Cancer | *Found not to affect diversity: Ineligibility for any trial* |
| Caldieraro-Bentley(51) | 2018 | USA | NR | NR | Older adults | Exercise studies | *Found not to affect diversity:*  *- Social marketing*  *- Community outreach*  *- Recruitment-site champions*  *- Recruitment-site education*  *- Health system recruitment*  *- Referrals paired with flyers* |
| Herrera(52) | 2010 | USA | Workshop | ~300 | Older adults | General | - Develop best practices and standardized protocols  - Establish and reinforce guidelines  - Use age friendly methods of communication  - Education and training for researchers  - Improve federal monitoring and accountability  - Reduce costs of participating for patients  - Address some of the damaging legacies of race/ethnicity |
| Jennings(53) | 2015 | UK | RCT | 1015 | Socially deprived and/or elderly | General | *Found not to improve diversity:*  *- Incentive* |
| Bellera(54) | 2013 | France | Systematic review | 87 RCTs | Older adults (>65 years) | Non-Hodgkins lymphoma | *Found not to improve diversity:*  *- Exclusion criteria that directly or indirectly exclude older adults*  *- Endpoints inappropriate for older adults* |
| Smorenburg(55) | 2014 | The Netherlands | Quantitative analysis | 1 trial; 260,700 replies | Older adults (>65 years) | Pneumonia vaccine | - Lower travel time to site  - Published advertorials in local newspapers  *Factors that did not improve diversity:*  *- Introduction letters on behalf of GPs prior to intervention letter* |
|  |  |  |  |  |  |  |  |
| **Women** | | | | | | | |
| Falcon(56) | 2011 | USA | Case study | 1 RCT; 287 individuals | Women, particularly of ethnic minority | HIV/AIDS | - Pretrial planning  - Inclusion/ exclusion criteria  - Site selection based on epidemiological trends  - Sites with strong community relationships, capacity for flexible office hours, and interest in and capacity to exceed standards of care for female patients  - Including representatives in investigator meetings  - Community advocates as consultants  - Recruitment targets  - Logo and branding chosen with target group  - Outreach toolkits  - Patient toolkits  - Acknowledgement certificates  - Birthday cards  - Clinic-based support groups  - Lunch and learns  - Home visits  - Individual reminders |
| Goldstein(57) | 2019 | USA | Review | 8 trials | Women | General | - Using administrative data for outcome assessment |
|  |  |  |  |  |  |  |  |
| **Other** | | | | | | | |
| Anderson(58) | 2017 | USA | Mixed-methods | 16 | Deaf individuals | Behavioural clinical trials | - Communication access, empathy, respect, strict conﬁdentiality procedures, trust, and transparency delivered in a sensory and linguistically appropriate way  - Team includes deaf and hearing members  - Variety of contact methods (video call and email)  - Materials in both American Sign Language video and written English  - Materials sent to deaf-related listservs, Facebook groups, and agencies that serve deaf individuals  - Present and network at relevant events  - Consent form written in plain English  - Consent available in American Sign Language  - Measures translated into ASL using translation, back-translation and equivalence comparison  - Extra assessment time  - Provide assessment results if requested  - Collect data in ASL form (e.g. video recording) |
| Hoffman(59) | 2019 | USA | Qualitative | 26 | HIV-infected individuals with opioid use disorder | HIV | - Trusted peer outreach/recruitment workers in the community  - Hospitalizations that offered windows of opportunities for screening and treatment induction  - Providing participant transportation  - Partnerships with harm reduction organisations for referrals |
| Siskind(60) | 2016 | USA | Case studies | 4 networks | Transgender people | HIV/AIDS | - Developing partnerships to build trust  - Input from transgender individuals on research plans and policies  - Standing advisory groups  - 2-step method of collecting sex at birth and gender identity on data collection forms  - Revised research protocol templates and policies for concept development  - Training for cultural sensitivity |
| Koziol-McLain(61) | 2016 | New Zealand | Mixed-methods | 501 | Women who experience violence | Safety decision aid | - Ads in online classified advertising platform (Jobs-Other-Volunteers)  - Registration website  - Friends and family referral to trial  *Factors that did not improve diversity:*  *- Print advertisements*  *- Networking*  *- Website links* |

Abbreviations: NR, Not Reported; RCT, Randomised Controlled Trial; UK, United Kingdom; USA, United States of Americ

1.	Zimmerman LP, Goel S, Sathar S, Gladfelter CE, Onate A, Kane LL, et al. A Novel Patient Recruitment Strategy: Patient Selection Directly from the Community through Linkage to Clinical Data. Appl Clin Inform. 2018;9(1):114-21.

2.	Brooks SE, Muller CY, Robinson W, Walker EM, Yeager K, Cook ED, et al. Increasing Minority Enrollment Onto Clinical Trials: Practical Strategies and Challenges Emerge From the NRG Oncology Accrual Workshop. J Oncol Pract. 2015;11(6):486-90.

3.	Ezeugwu CO, Laird A, Mullins CD, Saluja DS, Winston RA. Lessons learned from community-based minority health care serving system participation in an NIH clinical trial. J Natl Med Assoc. 2011;103(9-10):839-44.

4.	Heller C, Balls-Berry JE, Nery JD, Erwin PJ, Littleton D, Kim M, et al. Strategies addressing barriers to clinical trial enrollment of underrepresented populations: a systematic review. Contemp Clin Trials. 2014;39(2):169-82.

5.	Coakley M, Fadiran EO, Parrish LJ, Griffith RA, Weiss E, Carter C. Dialogues on diversifying clinical trials: successful strategies for engaging women and minorities in clinical trials. J Womens Health (Larchmt). 2012;21(7):713-6.

6.	Yu SH, Gumport NB, Mirzadegan IA, Mei YJ, Hein K, Harvey AG. Addressing the Challenges of Recruitment and Retention in Sleep and Circadian Clinical Trials. Behav Sleep Med. 2020;18(1):23-34.

7.	Boden-Albala B, Carman H, Southwick L, Parikh NS, Roberts E, Waddy S, et al. Examining Barriers and Practices to Recruitment and Retention in Stroke Clinical Trials. Stroke. 2015;46(8):2232-7.

8.	Ramsey TM, Snyder JK, Lovato LC, Roumie CL, Glasser SP, Cosgrove NM, et al. Recruitment strategies and challenges in a large intervention trial: Systolic Blood Pressure Intervention Trial. Clin Trials. 2016;13(3):319-30.

9.	Romero HR, Welsh-Bohmer KA, Gwyther LP, Edmonds HL, Plassman BL, Germain CM, et al. Community engagement in diverse populations for Alzheimer disease prevention trials. Alzheimer Dis Assoc Disord. 2014;28(3):269-74.

10.	Rooney LK, Bhopal R, Halani L, Levy ML, Partridge MR, Netuveli G, et al. Promoting recruitment of minority ethnic groups into research: qualitative study exploring the views of South Asian people with asthma. J Public Health (Oxf). 2011;33(4):604-15.

11.	Barnett J, Aguilar S, Brittner M, Bonuck K. Recruiting and retaining low-income, multi-ethnic women into randomized controlled trials: successful strategies and staffing. Contemp Clin Trials. 2012;33(5):925-32.

12.	Adams SA, Heiney SP, Brandt HM, Wirth MD, Khan S, Johnson H, et al. A comparison of a centralized versus de-centralized recruitment schema in two community-based participatory research studies for cancer prevention. J Community Health. 2015;40(2):251-9.

13.	Diehl KM, Green EM, Weinberg A, Frederick WA, Holmes DR, Green B, et al. Features associated with successful recruitment of diverse patients onto cancer clinical trials: report from the American College of Surgeons Oncology Group. Ann Surg Oncol. 2011;18(13):3544-50.

14.	Rivers D, August EM, Sehovic I, Lee Green B, Quinn GP. A systematic review of the factors influencing African Americans' participation in cancer clinical trials. Contemp Clin Trials. 2013;35(2):13-32.

15.	Wang JH, Sheppard VB, Liang W, Ma GX, Maxwell AE. Recruiting Chinese Americans into cancer screening intervention trials: strategies and outcomes. Clin Trials. 2014;11(2):167-77.

16.	Napoles A, Cook E, Ginossar T, Knight KD, Ford ME. Applying a Conceptual Framework to Maximize the Participation of Diverse Populations in Cancer Clinical Trials. Adv Cancer Res. 2017;133:77-94.

17.	Fischer SM, Kline DM, Min SJ, Okuyama S, Fink RM. Apoyo con Cariño: Strategies to Promote Recruiting, Enrolling, and Retaining Latinos in a Cancer Clinical Trial. J Natl Compr Canc Netw. 2017;15(11):1392-9.

18.	Sturgeon KM, Hackley R, Fornash A, Dean LT, Laudermilk M, Brown JC, et al. Strategic recruitment of an ethnically diverse cohort of overweight survivors of breast cancer with lymphedema. Cancer. 2018;124(1):95-104.

19.	Nickell A, Stewart SL, Burke NJ, Guerra C, Cohen E, Lawlor C, et al. Engaging limited English proficient and ethnically diverse low-income women in health research: A randomized trial of a patient navigator intervention. Patient Educ Couns. 2019;102(7):1313-23.

20.	Tabriz AA, Fleming PJ, Shin Y, Resnicow K, Jones RM, Flocke SA, et al. Challenges and opportunities using online portals to recruit diverse patients to behavioral trials. J Am Med Inform Assoc. 2019;26(12):1637-44.

21.	Regnante JM, Richie NA, Fashoyin-Aje L, Vichnin M, Ford M, Roy UB, et al. US Cancer Centers of Excellence Strategies for Increased Inclusion of Racial and Ethnic Minorities in Clinical Trials. J Oncol Pract. 2019;15(4):e289-e99.

22.	Cunningham-Erves J, Barajas C, Mayo-Gamble TL, McAfee CR, Hull PC, Sanderson M, et al. Formative research to design a culturally-appropriate cancer clinical trial education program to increase participation of African American and Latino communities. BMC Public Health. 2020;20(1):840.

23.	Wallington SF, Dash C, Sheppard VB, Goode TD, Oppong BA, Dodson EE, et al. Enrolling Minority and Underserved Populations in Cancer Clinical Research. Am J Prev Med. 2016;50(1):111-7.

24.	Selak V, Crengle S, Elley CR, Wadham A, Harwood M, Rafter N, et al. Recruiting equal numbers of indigenous and non-indigenous participants to a 'polypill' randomized trial. Int J Equity Health. 2013;12:44.

25.	Waheed W, Husain N, Allen G, Atif N, Aseem S, Waquas A, et al. Recruitment strategies for British South Asians in 5 depression trials: A mixed method study. J Affect Disord. 2015;185:195-203.

26.	Horowitz CR, Sabin T, Ramos M, Richardson LD, Hauser D, Robinson M, et al. Successful recruitment and retention of diverse participants in a genomics clinical trial: a good invitation to a great party. Genet Med. 2019;21(10):2364-70.

27.	Bass SB, D'Avanzo P, Alhajji M, Ventriglia N, Trainor A, Maurer L, et al. Exploring the Engagement of Racial and Ethnic Minorities in HIV Treatment and Vaccine Clinical Trials: A Scoping Review of Literature and Implications for Future Research. AIDS Patient Care STDS. 2020;34(9):399-416.

28.	Maar MA, Beaudin V, Yeates K, Boesch L, Liu P, Madjedi K, et al. Wise Practices for Cultural Safety in Electronic Health Research and Clinical Trials With Indigenous People: Secondary Analysis of a Randomized Clinical Trial. J Med Internet Res. 2019;21(11):e14203.

29.	Duda C, Mahon I, Chen MH, Snyder B, Barr R, Chiles C, et al. Impact and costs of targeted recruitment of minorities to the National Lung Screening Trial. Clin Trials. 2011;8(2):214-23.

30.	Andreae MH, Nair S, Gabry JS, Goodrich B, Hall C, Shaparin N. A pragmatic trial to improve adherence with scheduled appointments in an inner-city pain clinic by human phone calls in the patient's preferred language. J Clin Anesth. 2017;42:77-83.

31.	Tilley BC, Mainous AG, 3rd, Elm JJ, Pickelsimer E, Soderstrom LH, Ford ME, et al. A randomized recruitment intervention trial in Parkinson's disease to increase participant diversity: early stopping for lack of efficacy. Clin Trials. 2012;9(2):188-97.

32.	Goff SL, Youssef Y, Pekow PS, White KO, Guhn-Knight H, Lagu T, et al. Successful Strategies for Practice-Based Recruitment of Racial and Ethnic Minority Pregnant Women in a Randomized Controlled Trial: the IDEAS for a Healthy Baby Study. J Racial Ethn Health Disparities. 2016;3(4):731-7.

33.	Brown SD, Partee PN, Feng J, Quesenberry CP, Hedderson MM, Ehrlich SF, et al. Outreach to diversify clinical trial participation: A randomized recruitment study. Clin Trials. 2015;12(3):205-11.

34.	Burlew AK, Weekes JC, Montgomery L, Feaster DJ, Robbins MS, Rosa CL, et al. Conducting research with racial/ethnic minorities: methodological lessons from the NIDA Clinical Trials Network. Am J Drug Alcohol Abuse. 2011;37(5):324-32.

35.	Hughson JA, Woodward-Kron R, Parker A, Hajek J, Bresin A, Knoch U, et al. A review of approaches to improve participation of culturally and linguistically diverse populations in clinical trials. Trials. 2016;17(1):263.

36.	Amorrortu RP, Arevalo M, Vernon SW, Mainous AG, 3rd, Diaz V, McKee MD, et al. Recruitment of racial and ethnic minorities to clinical trials conducted within specialty clinics: an intervention mapping approach. Trials. 2018;19(1):115.

37.	Clark LT, Watkins L, Piña IL, Elmer M, Akinboboye O, Gorham M, et al. Increasing Diversity in Clinical Trials: Overcoming Critical Barriers. Curr Probl Cardiol. 2019;44(5):148-72.

38.	De las Nueces D, Hacker K, DiGirolamo A, Hicks LS. A systematic review of community-based participatory research to enhance clinical trials in racial and ethnic minority groups. Health Serv Res. 2012;47(3 Pt 2):1363-86.

39.	Ford ME, Siminoff LA, Pickelsimer E, Mainous AG, Smith DW, Diaz VA, et al. Unequal burden of disease, unequal participation in clinical trials: solutions from African American and Latino community members. Health Soc Work. 2013;38(1):29-38.

40.	Yamal JM, Robertson CS, Rubin ML, Benoit JS, Hannay HJ, Tilley BC. Enrollment of racially/ethnically diverse participants in traumatic brain injury trials: effect of availability of exception from informed consent. Clin Trials. 2014;11(2):187-94.

41.	Vander Stoep A, Myers K. Methodology for conducting the children's attention-deficit hyperactivity disorder telemental health treatment study in multiple underserved communities. Clin Trials. 2013;10(6):949-58.

42.	Kaur G, Smyth RL, Powell CV, Williamson P. A survey of facilitators and barriers to recruitment to the MAGNETIC trial. Trials. 2016;17(1):607.

43.	Le Rouzic MA, Claudot F. Characteristics of parental decision-making for children with advanced cancer who are offered enrollment in early-phase clinical trials: A systematic review. Pediatr Hematol Oncol. 2020;37(6):500-29.

44.	Sacheck JM, Van Rompay MI, Olson EM, Chomitz VR, Goodman E, Gordon CM, et al. Recruitment and retention of urban schoolchildren into a randomized double-blind vitamin D supplementation trial. Clin Trials. 2015;12(1):45-53.

45.	Fern LA, Lewandowski JA, Coxon KM, Whelan J. Available, accessible, aware, appropriate, and acceptable: a strategy to improve participation of teenagers and young adults in cancer trials. Lancet Oncol. 2014;15(8):e341-50.

46.	Lavender V, Gibson F, Brownsdon A, Fern L, Whelan J, Pearce S. Health professional perceptions of communicating with adolescents and young adults about bone cancer clinical trial participation. Support Care Cancer. 2019;27(2):467-75.

47.	Knopf AS, Ott MA, Liu N, Kapogiannis BG, Zimet GD, Fortenberry JD, et al. Minors' and Young Adults' Experiences of the Research Consent Process in a Phase II Safety Study of Pre-exposure Prophylaxis for HIV. J Adolesc Health. 2017;61(6):747-54.

48.	Wisk LE, Nelson EB, Magane KM, Weitzman ER. Clinical Trial Recruitment and Retention of College Students with Type 1 Diabetes via Social Media: An Implementation Case Study. J Diabetes Sci Technol. 2019;13(3):445-56.

49.	Freedman RA, Dockter TJ, Lafky JM, Hurria A, Muss HJ, Cohen HJ, et al. Promoting Accrual of Older Patients with Cancer to Clinical Trials: An Alliance for Clinical Trials in Oncology Member Survey (A171602). Oncologist. 2018;23(9):1016-23.

50.	Canouï-Poitrine F, Lièvre A, Dayde F, Lopez-Trabada-Ataz D, Baumgaertner I, Dubreuil O, et al. Inclusion of Older Patients with Cancer in Clinical Trials: The SAGE Prospective Multicenter Cohort Survey. Oncologist. 2019;24(12):e1351-e9.

51.	Caldieraro-Bentley AJ, Kelechi TJ, Treat-Jacobson D, Mueller M. Challenges in recruitment of persons with peripheral artery disease for exercise studies. J Vasc Nurs. 2018;36(3):111-20.

52.	Herrera AP, Snipes SA, King DW, Torres-Vigil I, Goldberg DS, Weinberg AD. Disparate inclusion of older adults in clinical trials: priorities and opportunities for policy and practice change. Am J Public Health. 2010;100 Suppl 1(Suppl 1):S105-12.

53.	Jennings CG, MacDonald TM, Wei L, Brown MJ, McConnachie L, Mackenzie IS. Does offering an incentive payment improve recruitment to clinical trials and increase the proportion of socially deprived and elderly participants? Trials. 2015;16:80.

54.	Bellera C, Praud D, Petit-Monéger A, McKelvie-Sebileau P, Soubeyran P, Mathoulin-Pélissier S. Barriers to inclusion of older adults in randomised controlled clinical trials on Non-Hodgkin's lymphoma: a systematic review. Cancer Treat Rev. 2013;39(7):812-7.

55.	Smorenburg AJ, Oosterman BJ, Grobbee DE, Bonten MJ, Roes KC. Effects of recruitment strategies and demographic factors on inclusion in a large scale vaccination trial in adults 65 years and older. Vaccine. 2014;32(25):2989-94.

56.	Falcon R, Bridge DA, Currier J, Squires K, Hagins D, Schaible D, et al. Recruitment and retention of diverse populations in antiretroviral clinical trials: practical applications from the gender, race and clinical experience study. J Womens Health (Larchmt). 2011;20(7):1043-50.

57.	Goldstein KM, Duan-Porter W, Alkon A, Olsen MK, Voils CI, Hastings SN. Enrollment and Retention of Men and Women in Health Services Research and Development Trials. Womens Health Issues. 2019;29 Suppl 1:S121-s30.

58.	Anderson ML, Wolf Craig KS, Ziedonis DM. Barriers and Facilitators to Deaf Trauma Survivors' Help-Seeking Behavior: Lessons for Behavioral Clinical Trials Research. J Deaf Stud Deaf Educ. 2017;22(1):118-30.

59.	Hoffman KA, Baker R, Kunkel LE, Waddell EN, Lum PJ, McCarty D, et al. Barriers and facilitators to recruitment and enrollment of HIV-infected individuals with opioid use disorder in a clinical trial. BMC Health Serv Res. 2019;19(1):862.

60.	Siskind RL, Andrasik M, Karuna ST, Broder GB, Collins C, Liu A, et al. Engaging Transgender People in NIH-Funded HIV/AIDS Clinical Trials Research. J Acquir Immune Defic Syndr. 2016;72 Suppl 3(Suppl 3):S243-7.

61.	Koziol-McLain J, McLean C, Rohan M, Sisk R, Dobbs T, Nada-Raja S, et al. Participant Recruitment and Engagement in Automated eHealth Trial Registration: Challenges and Opportunities for Recruiting Women Who Experience Violence. J Med Internet Res. 2016;18(10):e281.
